# Acceptance of Innovative Food Among Tourists: Psychological Factors and Generational Differences in the Post-Transition Context of Serbia

**DOI:** 10.3390/foods14213607

**Published:** 2025-10-23

**Authors:** Tamara Gajić, Dragan Vukolić, Snežana Knežević, Ana Spasojević, Filip Đoković, Srđan Milošević, Mladen Radišić, Maja Radišić, Dušan Pevac

**Affiliations:** 1 Geographical Institute “Jovan Cvijić”, Serbian Academy of Sciences and Arts, Đure Jakšića 9, 11000 Belgrade, Serbia; tamara.gajic.1977@gmail.com; 2 Faculty of Organizational Studies Eduka, University of Business Academy in Novi Sad, Majke Jevrosime 15, 11000 Belgrade, Serbia; fdjokovic@vos.edu.rs (F.Đ.); srdjan.milosevic@vos.edu.rs (S.M.); 3 Faculty of Tourism and Hotel Management, University of Business Studies, Jovana Dučića 23a, 78000 Banja Luka, Bosnia and Herzegovina; 4 Academy of Applied Studies Polytechnic, 11000 Belgrade, Serbia; lesta59@yahoo.com; 5 Faculty of Economics, University of Kragujevac, 34000 Kragujevac, Serbia; ana.spasojevic1985@gmail.com; 6 Department of Industrial Engineering and Management, Faculty of Technical Sciences, University of Novi Sad, 21000 Novi Sad, Serbia; mladenr@uns.ac.rs (M.R.); dusan@foodscalehub.com (D.P.); 7 Foodscale Hub, 21000 Novi Sad, Serbia; maja.radisic@polj.edu.rs; 8 Department of Agricultural Economics and Rural Sociology, Faculty of Agriculture, University of Novi Sad, 21000 Novi Sad, Serbia

**Keywords:** innovative food, gastronomic tourism, neophobia, involvement in food, generational differences, tourist behavior

## Abstract

The readiness of tourists to accept innovative food is investigated in this research through the prism of the Protection Motivation Theory and the Theory of Planned Behavior, combining two previously developed yet seldom researched psychological dimensions, namely, food neophobia as a restraining force and food involvement as a motivating force. The quantitative approach and the generation-by-generation analysis using partial least squares (PLS-SEM) and multiple group analysis were used to conduct the study on a sample of 985 domestic tourists in Serbia. The results suggest that food involvement eases openness toward gastronomic innovations and mitigates the negative impact of neophobia, whereas the generational differences reveal that younger tourists are more willing to be experimental, and older generations tend to be conservative in their food consumption. The study is relevant to the academic literature because it puts motivational and barrier factors into context within the PMT and TPB paradigms and provides operational implications for the design of tourism propositions that can be used to promote innovative and sustainable gastronomic experiences. The novelty of the present study is that it uses the hybrid model of food neophobia and food involvement in the generational context of a post-transition society, i.e., Serbia.

## 1. Introduction

The modern tourism and hotel industry is facing the challenges of international transformations in which gastronomy is taking shape as one of the key dimensions of the tourist experience [1,2]. Although innovative food is often seen as a means of increasing the value of the destination, the existing literature indicates the presence of pronounced contradictions. Yang et al. [3] confirm that, in addition to the positive effects of the introduction of gastronomic innovations, unwanted consequences may arise, such as an increase in the amount of food waste and excessive consumption. On the other hand, Shah et al. [4] highlight the discrepancy between expressed intentions and actual consumer behavior, which calls into question the ability of standard models to fully explain the process of adoption of innovations in tourism. Such contradictions are particularly pronounced in terms of cultural and generational differences, which have been insufficiently elaborated in previous research [5,6,7]. They are given additional complexity by psychological factors that shape the behavior of tourists. Hossain et al. [8] state that satisfaction and perception of innovation are the most reliable predictors of behavior but do not explain how different generations perceive gastronomic novelties. Zhan et al. [9] indicate that openness to innovation cannot be observed without taking into account individual preferences, especially among Generation Z, while the phenomenon of neophobia is rarely considered in the tourism context.

Such findings point to the importance of connecting psychological barriers and motivational processes that influence the willingness of tourists to accept innovative forms of food. Starting from the Theory of Protection Motivation and the Theory of Planned Behavior, the aim of this research is to examine the influence of individual psychological characteristics, specifically neophobia and involvement in food, on the willingness of tourists to accept innovative food, with a special focus on generational differences between Generation Z and baby boomers. The choice of these two cohorts is based on their contrasting value systems: while Generation Z shows a high degree of curiosity, technological savvy, and willingness to experiment, baby boomers are more inclined to traditional patterns of behavior and a lower level of readiness for change [3]. Other generations (X and Y) were not included in order to avoid overlapping middle cohorts and to provide a clearer comparison of the final generational groups, which is a deliberate methodological limitation that is planned to be overcome in future research.

Unlike previous studies that emphasize the technological aspects of innovations in tourism, the novelty of this research is reflected in the study of cultural and experiential innovations in gastronomy as indicators of the tourist experience. Thus, gastronomy is seen not only as a culinary product but also as a cultural and identity symbol. The research was conducted among domestic tourists in Serbia using a quantitative approach and modeling using partial least squares (PLS-SEM), with multiple group analysis (MGA) by generation. It is expected that neophobia has a negative effect and food involvement has a positive effect on the acceptance of innovative gastronomic experiences, with more pronounced effects of neophobia in the elderly and a greater impact of involvement in younger respondents.

The theoretical contribution of the paper is reflected in the integration of motivational and barrier psychological dimensions within the Protection Motivation Theory (PMT) and the Theory of Planned Behavior (TPB), thus extending the existing models to the domain of gastronomic tourism, an area in which these theories have not been systematically applied so far. Such an approach enables a deeper understanding of the mechanisms that link risk perception, motivation, and behavioral intention of tourists in accepting food innovations. The scientific significance of the work lies in the conceptual model that, for the first time, integrates neophobia and involvement in food as complementary predictors, opening the possibility to formulate a universal theoretical framework applicable in different cultural and post-transition contexts. The practical contribution is reflected in the development of guidelines for the design of the gastronomic offer that connects innovative and sustainable practices, adapted to the specific values and behavioral patterns of different generational segments of tourists.

## 2. Theoretical Background

### 2.1. Protection Motivation Theory (PMT) and Theory of Planned Behavior (TPB)

This study rests on two theoretical concepts, which are complementary to each other: Protection Motivation Theory (PMT) and Theory of Planned Behavior (TPB). The evolution of behavior can be explained by the Theory of Protection Motivation [10,11], which contains the evaluation of risk and the personal competencies to cope with the risk. In this approach, the balance between the threat and the control is the determinant of behavior. Food neophobia in relation to gastronomy may be viewed as an element of the PMT protection mechanism, according to which not wanting to eat something strange is a means of perceived risk reduction and ensuring psychological safety. Conversely, the Theory of Planned Behavior [12] relies on the point that behavior is a product of intention, and the development of intention depends on attitude to behavior, subjective norms, and perceived behavioral control. These factors are important in the tourism context in regard to looking at the choices of travelers to adopt new gastronomic experiences.

This study has developed a dual-pronged theoretical concept that connects inhibitory processes with motivational processes by combining the PMT and TPB frameworks. The theoretical constructs of risk avoidance and formation of behavioral intention are operationalized using the constructs of neophobia (as a constraining factor) and food involvement (as a motivating factor). This type of integration will make both theories applicable to the sphere of gastronomic tourism, which, to date, has not been systematically utilized, and will allow the mechanisms that interrelate risk perception, motivation, and behavioral intention of tourists during the acceptance of gastronomic innovations to be better understood. Moreover, this theoretical framework shows intergenerational variations in the responses to dietary novelties because various generations define attitudes concerning risk, tradition, and experimentation differently. Therefore, not only are the models of PMT and TPB applied in the research, but a new conceptual model is also formed, which links psychological barriers and motivational drivers in the acceptance of innovative food in tourism.

### 2.2. Acceptance of Innovative Food by Tourists

Authors Monaco and Sacchi [13] indicate that the metaverse can serve as a space to reduce perceived risk when encountering new types of food. Their contribution lies in linking gastronomic innovations with digital experiences that can increase tourists’ willingness to accept new products, although a limitation is the fact that such practices have not yet been validated in real contexts. In their research, An et al. [14] show that the perceived benefits and ease of use of intermediary services strongly shape the intention to consume new types of food available through digital channels. Their contribution is in confirming the importance of the perception of usefulness and simplicity, but the weakness is that they emphasize the mediation of technology, while the food itself, as innovative tourist content, remains in the background. Berakon et al. [15] extend this approach to halal tourism and emphasize that religious and cultural factors can significantly shape tourists’ willingness to accept new food products. Their contribution is the integration of cultural dimensions, while their weakness remains the neglect of individual differences in consumer attitudes. Shukri et al. [16] focus attention on the fact that the willingness to try new food depends on the perception of risk and individual attitudes, whereby tourists who are more risk-tolerant show a greater willingness to innovate. Their contribution is an understanding of the psychological mechanisms of acceptance of innovative foods, although a limitation is the local context, which makes it difficult to generalize the findings. Laureati et al. [17], in their systematic review, confirm that trust in institutions, food safety, and the perception of sustainability play a key role in the acceptance of new food products. Their contribution is to strengthen the link between innovation and sustainability, but their weakness is that they remain at a general level without a direct link to tourism behavior.

Khan and Mehmood [18] point out that resistance to the introduction of new food practices may arise from negative attitudes of employees, but the weakness of their work is that tourists, as the primary users of new products, remain neglected. Khan et al. [19] introduce environmental awareness as a significant factor in the acceptance of innovative solutions such as edible packaging, showing how sustainability can be a powerful motivator. A weakness is that individual psychological differences are ignored, thus opening up space for constructs such as neophobia or food involvement. Samaddar and Mondal [20] emphasize that balancing traditional and innovative dishes encourages responsible tourism behavior and contributes to sustainable consumption. Their contribution is a conceptual framework for the integration of authenticity and innovation but without empirical confirmation. Wang [21] shows that the individual innovativeness of tourists affects the way they perceive the authenticity of new food formats, such as food truck offerings. His research confirms the importance of individual psychological factors, although it remains limited to one specific segment. Amiri et al. [22] confirm that tourists’ openness to innovation and their psychological readiness significantly shape continuity in accepting new forms of food, including self-service formats. Their contribution is emphasizing the role of psychological factors, while the weakness remains a limited focus on the way of serving instead of the innovative food itself. The joint findings of these studies show that the acceptance of innovative food among tourists depends on a combination of individual psychological characteristics, cultural, and contextual factors, which justifies our decision to focus the analysis on specific constructs related to gastronomy and tourist behavior.

### 2.3. Individual Factors in the Acceptance of Innovative Food and Differences Between Generations

Acceptance of innovative food among tourists is shaped by the relationship between psychological barriers and motivational drivers. The Food Neophobia Scale (FNS), developed by Pliner and Hobden [23], is a measure of resistance to unfamiliar food and is often identified as a key barrier to adopting new gastronomic experiences. Chen et al. [24] show that a higher level of neophobia significantly reduces tourists’ willingness to try innovative products, while Cifci et al. [25] emphasize that tourists with pronounced neophobia avoid street food experiences more often and exhibit a lower level of positive post-travel attitudes. These findings suggest that neophobia acts as an inhibitory factor in the tourist experience, which is why, in this paper, we hypothesize that greater neophobia will reduce tourists’ willingness to accept innovative food.

**H1.**  *Food Neophobia has a negative impact on tourists’ willingness to accept innovative food.*

In contrast, the Food Involvement Scale (FIS) [26] measures the degree of personal involvement in food and can be seen as a motivating factor. Laureati et al. [17] confirm that high involvement stimulates curiosity and facilitates the acceptance of new products, while Cheriyan et al. [27] emphasize that balancing traditional and innovative dishes contributes to responsible and sustainable consumer behavior. In the tourism context, research shows that tourists with a higher degree of involvement demonstrate a greater willingness to try the innovative gastronomic offers of destinations [28]. Based on these findings, we propose the following hypotheses:

**H2.**  *Food involvement has a positive influence on tourists’ willingness to accept innovative food.*

**H3.**  *Food involvement moderates the negative effect of neophobia on innovative food acceptance.*

It has always been empirically recorded that the impact of neophobia and participation is not the same across generational lines. According to Ding et al. [29,30], the younger generation (Generation Z) is more willing to adopt new gastronomic habits, whereas the older generations are still more likely to use more traditional consumption habits. The same differences are substantiated by Hoang et al. [31], who note that Gen Z and Gen Y consider the experiential and social elements when selecting traditional food in restaurants, and adherence to tradition proves to be the more conservative choice pattern in older generations. Poyoi et al. [32] also shed some light, suggesting that young consumers (particularly Gen Z) value food sharing and social reasons more, which means that they are more ready to use food as part of a more extended innovation experience. Conversely, a study conducted on the Baby Boomer generation indicates that their culinary choices usually tend to be influenced by culture and habits that they have learned. Sui et al. [33] demonstrated that among older consumers, such as Boomers, attitudes toward green food highly rely on food literacy and attitudes toward social norms, which justifies the interrelation between neophobia and the dependence on familiar behavioral patterns. The same tendency was traced by Wiangkham et al. [34], who demonstrate that Baby Boomers are less inclined to accept virtual reality in tourism than Gen Z, which can also be applied to the sphere of gastronomy, where innovations are harder to accept when they break existing patterns. On the contrary, Akoğul [35] makes it clear that Gen Z tourists report being more inclined toward sustainable local food, meaning that they are willing to relate innovation to sustainability and destination identity. Such data prove that younger generations tend to experiment more and bring food closer to social, digital, and sustainable habits, whereas older generations are more connected to tradition and institutional trust. The disadvantage of the majority of these studies, however, is that they deal with particular segments of consumption (e.g., traditional food, green food, VR tourism) without marking out the psychological concepts like neophobia and food involvement [36,37]. This leaves our work open, where we hypothesize that the negative influence of neophobia on the acceptance of innovative food will be stronger among Boomers, and the positive influence of food involvement will be stronger among Gen Z.

**H4.**  *The influence of the tendency towards food neophobia on the acceptance of innovative food will be more pronounced among members of the Baby Boomers generation than among members of the Z generation.*

**H5.**  *The influence of food involvement on the acceptance of innovative food will be more pronounced among members of Generation Z than among members of the Baby Boomer generation.*

### 2.4. Theoretical Justification and Model Development

The combination of the Food Neophobia Scale (FNS) and the Food Involvement Scale (FIS) provides new analytical value in studying the acceptance of innovative foods in tourism because it allows for a balanced view of the limiting and enabling factors of consumer behavior. While the FNS is aimed at identifying barriers such as fear, aversion, or resistance to new foods [38,39], the FIS emphasizes the active engagement and involvement of individuals in the selection, preparation, and consumption of food, which acts as a strong driver of the willingness to adopt new gastronomic experiences [40]. It is this dichotomy that makes this model particularly relevant for the tourism context, where different cultures, tastes, and expectations meet.

Previous research has shown that neophobia can significantly limit choice and reduce tourists’ willingness to try innovative foods [41,42], while tourists with higher levels of food involvement are more likely to experiment and connect gastronomy with the cultural and experiential values of the destination [43,44]. This suggests that the FNS and FIS are not contradictory but complementary scales that together explain the full spectrum of behavior, from resistance to acceptance. In addition, research such as Zhang et al. [45] shows that the balance between the familiar and the new shapes the gastronomic tourism experience, which directly links to the concept of neophobia and neophilia. At the same time, Laureati et al. [17] and Naderi et al. [46] emphasize that the acceptance of innovative foods is not only a matter of personal preference but also has a broader impact on sustainability, as it encourages the development of environmentally friendly and socially responsible food systems. Tourism destinations that manage to balance these factors can contribute to waste reduction, greater resource efficiency, and the preservation of cultural diversity [47].

**Figure 1:**
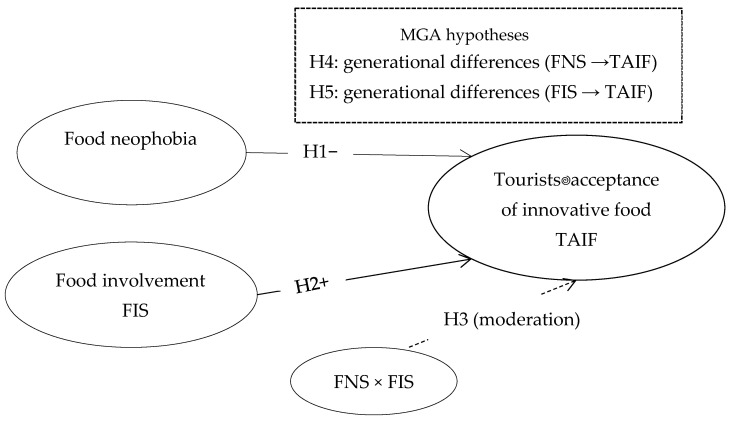
Conceptual model. Note: H4 and H5 refer to generational differences and are tested through Multi-Group Analysis (MGA); therefore, they are not displayed as direct paths in the structural model.

In this context, the proposed research model combines FNS and FIS and applies them to a multigenerational sample (Gen Z and Boomers) to determine how different ages and cultural orientations influence the acceptance of gastronomic innovations in a tourism environment. This fills a clear gap in the literature, as previous attention has been fragmented, either focusing exclusively on neophobia [41,48] or on aspects of involvement and consumption value [40,49,50]. Based on previous literature, a theoretical framework was built that integrates constraints and incentives and allows for hypothesis testing through structural modeling (SEM) and multigroup analysis (MGA). This approach not only allows for the examination of the individual effects of FNS and FIS but also for the comparison of their impact across generations. The particular value of the work lies in the clear connection of these scales with the concept of TAIF (Tourist Acceptance of Innovative Food), thus providing a methodologically and theoretically grounded model that balances both resistances and drivers [51,52,53]. This creates a robust framework for interpreting the acceptance of innovative food in tourism, which represents an original contribution to the literature and practical strategies in the field of gastronomic and sustainable tourism (Figure 1).

## 3. Methodological Framework

### 3.1. Empirical Context and Sample Characteristics

**Figure 2:**
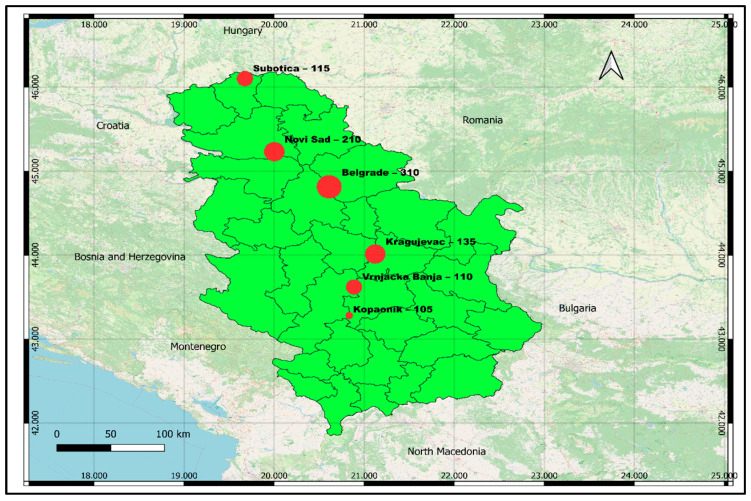
Research area (Source: Authors, created in QGIS v. 3.34.9).

The research is based on data collected from domestic tourists in Serbia, reflecting their perceptions and attitudes toward innovative food in a national context, in the period from December 2024 to June 2025. In major cities and destinations with developed tourist and gastronomic potential: Belgrade (310), Novi Sad (210), Kragujevac (135), and Subotica (115), while Vrnjačka Banja (110) and Kopaonik (105) were included as important resorts and centers of tourism. Such a choice of locations enables coverage of urban areas with intensive gastronomic innovations, but also destinations that attract domestic tourists through wellness and recreational facilities. In this way, the heterogeneity of the sample and better external validity of the findings were ensured because different patterns of consumer and tourist behavior were included (Figure 2).

The decision to focus on domestic tourists is justified by the fact that they represent a key segment of the market during periods of instability in the country for the last eight months (starting from November 2024), when the number of international arrivals has visibly decreased, and at the same time, they best reflect the dynamics of acceptance of innovations in nutrition within the national context. Their inclusion contributes to the validity of the research because it allows for the observation of specific cultural and generational patterns regarding the acceptance of innovative food. The research was conducted using a survey method through a structured questionnaire. The survey was distributed on-site in hotels, restaurants, and tourist centers of the destinations. The research was conducted anonymously, with clear information about the aim and scope of the study, as well as the possibility of voluntary withdrawal. The CAPI (Computer-Assisted Personal Interviewing) method was used for data collection, with the interviewers using tablets to ensure accuracy and efficiency in entering answers. A priori G*Power analysis (version 3.1.9.7) [54] (F-test, multiple regression; f^2^ = 0.02, α = 0.05, 1–β = 0.95, 6 predictors) showed that a minimum sample of ≈650 was required, while a sample of 985 subjects was achieved (acceptable thresholds: small effect f^2^ ≥ 0.02, power 1–β ≥ 0.80). For MGA (*t*-test, d = 0.25, α = 0.05, 1–β = 0.95, unequal groups), the minimum sample is ≈650, which is also exceeded (acceptable thresholds: small effect d ≥ 0.20, power 1–β ≥ 0.80). Sensitivity shows that with a sample of 985 respondents, we detect effects of size f^2^ ≈ 0.014 in the regression and d ≈ 0.19 between groups, which is below the limit of a small effect, confirming the adequate and high power of the research.

The generational categorization used in the given research is based on the recent findings of tourism and consumer behavior research [8,9] and the generational boundaries outlined by the Pew Research Center [55]. In this regard, Generation Z entails those who were born from 1995 to 2010, and Baby Boomers encompass those who were born from 1946 to 1964. To avoid overlapping cohorts and to have a distinct contrast between the youngest and oldest segments of the generations, Generations X (1965–1980) and Y/Millennials (1981–1994) were purposely omitted. This choice is in line with the conceptual purpose of the research, which aims to embrace the polarized behavioral tendencies between cohorts that are most receptive to innovativeness and those that are most wedded to conventional consumption behavior (Table 1).

Gen Z and Boomers were chosen as the research sample, and the proportion of each to the total number of participants was 48.9% and 51.1%, respectively, which was almost equal. There are two reasons why this structure of the sample is justified. The research was aimed at investigating the appeal of innovative food to tourists of various generations, and therefore it was essential to have an adequate number of respondents from both generations since this would make it possible to draw comparative conclusions. A balanced sample enhances the statistical strength of the tests and minimizes the chances of a lack of objectivity. These generations have been chosen based on their various roles in tourism and gastronomy. Gen Z is viewed as the carrier of new tendencies and exploration of food novelties, whereas Boomers are a large customer segment that is more attached to tradition and has greater purchasing capacity. Also, the fact that the respondents are heterogeneous in their gender, education, monthly income, and type of preferred travel is a factor that makes the findings valid since the sample represents actual social and tourist trends in Serbia. Through this, the representativeness and reliability of the results are guaranteed, which justifies the relevance and scientific value of the research. It is nearly homogeneous in the gender structure (50.7% and 49.3% male and female respondents). The slight disparities in the size of the subsamples of the analyses are due to the use of the listwise deletion procedure, which removed the respondents whose answers were missing or incomplete in the further analyses. The sample size was the same (*N* = 985), whereas the number of valid cases per generational group varied slightly owing to the completeness of the answers to individual statements of the construct. This method was used to guarantee the statistical stability and reliability of the model, as would be performed in the case of standard procedures of SEM analysis.

Most of the respondents completed higher or university education (58.5%), while a smaller share finished secondary school (35.9%), and only a few hold a doctoral degree (5.6%). The distribution of incomes shows that most respondents earn up to 1000 per month (86.9%), whereas only 13.1% earn above that amount. The status of employment is generational: Gen Z is characterized by students and employees, whereas Boomers are represented mostly by retirees. When it comes to travel goals or preferences, the most frequently selected areas are city tourism (44.2%) and mountain destinations (29.4%), while the least frequently selected area is wellness and spa tourism (26.4%) (Table 2).

To confirm the correctness of the approach to the methodology of the instrument, a pilot study was carried out on a sample of 52 domestic tourists in Belgrade and Novi Sad. The results obtained depicted that none of the items were incomprehensible and did not bring about dilemmas for the respondents. Statistical tests ensured that there was a good state of internal consistency. The Food Neophobia Scale (FNS), Food Involvement Scale (FIS), and the construct Tourist Acceptance of Innovative Foods (TAIF) had Cronbach’s α of 0.82, 0.85, and 0.88, respectively. The results of the reliability analysis using composite reliability (CR) demonstrated a range of 0.84–0.90, which is above the recommended value of 0.70, whereas the average variance extracted (AVE) score was between 0.52 and 0.63, which validates convergent validity. In addition, the values of the factor loadings in each of the cases were above the limit of 0.60, and no item had to be dropped. On the basis of these findings, it was concluded that the instrument is highly stable and measurement-reliable, and therefore it was completely used in the main research. The ethics of the research were maintained by making sure that the principle of informed consent was adhered to, such that all the participants were fully informed of the purpose of the research, the procedure, and the voluntary nature of participation. No data on the respondents that was personal or identifying were gathered, and this guaranteed total anonymity and privacy of the respondents. The data were only handled in aggregate form and without the possibility of identifying individual responses. By doing so, moral hazard was minimized to the least, and no risk was associated with the respondents in taking part in the research.

Even though the study strategically targets Generation Z and Baby Boomers to represent the difference between the youngest and oldest consumer groups, it is understood that the omission of Generation X (1965–1980) and Y/Millennials (1981–1994) makes the generational diversity range narrower. Such a decision was determined by methodological clarity and the desire to eliminate overlapping life stages and patterns of behavior characteristic of middle cohorts. Still, we know that Millennials and Generation X are crucial markets in the hospitality industry; they are economically active, and their purchasing power is high, as well as their attitude toward innovation and tradition is balanced. Thus, the next research will extend the generational framework and consider these groups, which will allow consideration of the development of variations in the acceptance of innovative food among the entire adult population in Serbia in greater detail.

### 3.2. Constructs and Measurement

The research was focused on the Food Neophobia Scale (FNS) and the Food Involvement Scale (FIS) because they are the most relevant and frequently used constructs in the literature on new food acceptance [23,26]. Both constructs have high validity and reliability, which allows precise measurement of key dimensions: resistance (neophobia) and motivation (involvement). Although the term “innovative food” in this research refers to dishes that are familiar to domestic tourists but prepared, served or combined in a new way, this approach still includes a certain degree of unfamiliarity in taste, form or composition. Therefore, the application of the Food Neophobia Scale (FNS) is justified, because even the smallest deviation from the usual sensory and cultural expectations can cause psychological resistance to the novel. In this research, examples of completely unknown or exotic dishes were not used, because the goal was to examine the reaction of tourists to realistic innovations in domestic gastronomy. However, future research could include examples of foreign or exotic foods to examine whether the degree of food unfamiliarity affects the intensity of neophobia and willingness to accept innovations.

Including a large number of variables in one SEM model at the initial research stage could reduce the visibility of the results and make it difficult to interpret the relationships. Therefore, the principle of model parsimony was applied, focusing on two basic psychological dimensions that offer a clear basis for hypothesis testing, while other characteristics (cultural curiosity, emotional intelligence, risk perception, attitudes towards sustainability) were left for further research. Before starting to fill out the questionnaire, the interviewers gave the participants a brief explanation of the meaning of the term “innovative food” within the scope of this research. Due to the fact that the meaning of such a term as innovative food is to be understood in a consistent way, all interviewers were given a standardized instruction sheet with a specific verbal definition and examples. The pilot study confirmed this definition, and no respondent indicated that they were unsure or confused about the meaning of the concept. The term was thus conceptualized as a culturally and experience-based approach to gastronomy in terms of familiar dishes being revisited using new combinations, presentation, or the introduction of sustainable and creative constituents, and not technological or industrial food innovations. It was stated that this means dishes that are basically familiar to domestic tourists but prepared, served, or combined in a new way (plant-based variants of traditional dishes, fusion cuisine, or the introduction of edible packaging). In this way, a common understanding of the concept was provided that is fully aligned with the constructs measured by the Food Neophobia Scale (FNS) and the Food Involvement Scale (FIS), which examine psychological barriers and motivational factors in the acceptance of new foods. All attitudes were rated on a seven-point Likert scale (1 = strongly disagree, 7 = strongly agree), which allowed precise quantitative measurement of individual differences.

The Food Neophobia Scale [23] measures the degree of consumer resistance to new and unfamiliar foods. This instrument has been used several times in gastronomy and tourism research because it enables the identification of obstacles to the acceptance of innovative dishes. In the context of tourism, FNS proves to be a key indicator of barriers when introducing new culinary products, which is particularly important for hotels and restaurants that develop plant-based or fusion menus. Although some authors point to the possibility of isolating subfactors (e.g., resistance to ethnic food or to food produced by new technologies), in most modern works, FNS is treated as a unique latent dimension that expresses a general reluctance to try new tastes. The Food Involvement Scale [26] assesses the degree of personal involvement and importance that an individual attaches to food in everyday life. Originally developed as a multidimensional scale with 12 statements, in food tourism research the FIS is often used in abbreviated versions and viewed through a single dimension of general food involvement. Such a methodological justification is warranted because it conforms to the principle of model parsimony in SEM, which permits a straightforward discovery of motivational impacts without any overlapping areas of subdimensions of purchase, preparation, and consumption. Even though the multidimensional nature of the original scale provides more benefits in terms of behavioral detailing, the unidimensional form is useful in terms of the overall psychological involvement with food, which is the most significant in the research of innovative food acceptance in the context of tourism. This model can, however, be extended in future research by including a complete multidimensional FIS structure to study the different stages of food-related behavior more thoroughly. Such an approach is methodologically justified because it emphasizes the central role of food in the tourist experience, regardless of the specific stages of purchase, preparation, or consumption [56]. In this research, FIS serves as an indicator of positive motivation, as tourists with a higher level of involvement are more likely to accept innovative gastronomic experiences. The combination of FNS and FIS in the same research framework is not contradictory but complementary [57].

While the FNS identifies the negative dimension of behavior, resistance and avoidance of new tastes, the FIS emphasizes the positive dimension: the enthusiasm and importance that an individual attaches to gastronomic experiences [58]. In this way, it is possible to analyze more complex patterns of behavior: a tourist can be highly involved in gastronomy but at the same time show a certain degree of neophobia. This is precisely why these two scales can be used together in empirical works. For example, Choe and Kim [59] emphasize that the combined use of FNS and FIS allows a better understanding of tourists’ willingness to try local food, while Mak et al. [60] show that high food involvement, combined with low levels of neophobia, is the strongest predictor of acceptance of new gastronomic experiences.

In addition to these two scales, a separate set of items for the latent variable Tourist Acceptance of Innovative Foods (TAIF) was developed within the research. This constructed instrument is based on the works of Ajzen [12] on the Theory of Planned Behavior (TPB) and Kim et al. [61] in the field of tourist gastronomy. TAIF measures tourists’ willingness to try, accept, and recommend innovative dishes during travel. Thus, the research model includes three key latent dimensions: neophobia, involvement in food, and acceptance of innovative food, which enables empirical testing of the influence of individual attitudes, barriers, and motivations on the formation of tourist intentions and behavior in catering (Table 3).

### 3.3. Analytical Procedures

Data analysis was carried out using the SmartPLS 4 software, within the Partial Least Squares Structural Equation Modeling (PLS-SEM) approach, while SPSS 25 was used for basic descriptive statistics. The research procedure was carried out through several stages that ensured a systematic check of the measurement and structural model.

In the first phase, a preliminary factor analysis was conducted in order to verify the basic structure of the newly formed Tourist Acceptance of Innovative Foods (TAIF) construct. The results of Kaiser–Meyer–Olkin (KMO) and Bartlett’s test of sphericity confirmed that the data meet the conditions for factor analysis, while the value of the eigenvalue (eigenvalue > 1) indicated a clear one-dimensional structure of the construct. This justifies its inclusion in further SEM validation.

In the second phase, the measurement model was validated within the PLS-SEM approach. The parameters of outer loadings were evaluated, with all values exceeding the recommended threshold of 0.70, which confirms the reliability of individual indicators. The reliability of the constructs was checked through the Cronbach α coefficient, rho_A and composite reliability (CR), with values greater than 0.70, which indicates a high internal consistency of the scales. Convergent validity was confirmed by average variance extracted (AVE), where all values were above the threshold of 0.50 [62,63]. Discriminant validity was examined using the Fornell–Larcker criteria and HTMT indicators, which remained below the recommended limit of 0.85. Globally, the model showed a good measure of fit through SRMR values that were below the recommended threshold of 0.08. This provided a complete assessment of the measurement model within the PLS-SEM framework, without the need for additional CFA analyses in the CB-SEM context [64].

The third phase was related to the validation of the structural model. Internal collinearity was checked, and it was determined that all VIF coefficient values remain below the threshold of 3.3, which eliminates the problem of multicollinearity among predictors. Significance and strength of paths (β coefficients) were tested using a bootstrapping procedure with 5000 resampled samples, while effect size (f^2^), explained variance (R^2^) and predictive relevance (Q^2^, assessed by the blindfolding technique) further shed light on the stability and predictive power of the model. Additionally, the predictive ability of the model was assessed by the PLSpredict procedure, which compares the PLS predictions to the corresponding linear benchmark model and provides an out-of-sample estimate of predictive relevance [65,66,67,68].

In the final stage, the moderation and multigroup effect was analyzed. The moderating effect was examined through the interaction between neophobia (FNS) and food involvement (FIS) in predicting acceptance of innovative foods (TAIF). The interaction term FNS × FIS was constructed in the program SmartPLS 4 using the Two-Stage approach method with the application of the orthogonalization procedure, which reduced the risk of multicollinearity. The significance of the interaction coefficient was assessed by a bootstrap procedure with 5000 resampled samples, which provides stable estimates of standard errors and *p*-values. In addition, the predictive power of the model with and without interaction was compared through ΔR^2^ and Stone–Geisser’s Q^2^ (blindfolding), while the size of the moderation effect was expressed through the f^2^ metric. Additionally, the MICOM (Measurement Invariance of Composite Models) test was conducted to check measurement invariance across generational groups. The results confirmed the configural invariance through identical model specification and the compositional invariance through the permutation test (*p* > 0.05), as well as the equality of mean values and variances (*p* > 0.05). This fulfills the conditions for the reliable implementation of multigroup analysis (MGA) [69,70,71].

## 4. Findings

### 4.1. Descriptive Outcomes and Factor Validation

Since the Food Neophobia Scale (FNS) and Food Involvement Scale (FIS) have previously been validated multiple times in the literature, there was no need to conduct an exploratory factor analysis (EFA) on these constructs. However, for the adapted Tourists’ Acceptance of Innovative Food (TAIF) scale, preliminary EFA (promax rotation) was conducted to confirm the underlying factor structure. The results of the Kaiser–Meyer–Olkin test (KMO = 0.892) and Bartlett’s test of sphericity (χ^2^(105) = 1523.674, *p* < 0.001) confirmed the suitability of the data for factor analysis. The analysis singled out one factor with eigenvalue = 1.433, which explains 9.6% of the total variance, which clearly indicates the unidimensionality of the TAIF construct. All factor loadings were above 0.70, which confirmed the internal consistency and justification for including TAIF in further PLS-SEM validation.

In the construct of the Food Neophobia Scale (FNS), the means were a little lower than the center of the scale (m ≈ 3.8–4.0), which suggested a moderate predisposition of the tourists to avoid new and unfamiliar foods. The standard deviations were in the middle (≈1.1), and it means that there are certain discrepancies, but not extremely big ones. Outer loadings were all above the necessary threshold of 0.70 (0.734–0.783), which will ensure that all the items are consistent predictors of the latent construct and that the FNS measures what is expected. In the case of the Food Involvement Scale (FIS), the average scores were slightly higher (m ≈ 5.0–5.3), which means that tourists have a high level of food involvement in their lives and that gastronomy is also an important part of their lives and experiences. Standard deviations were not high (1.0–1.2), and the external loadings were between 0.706 and 0.762, hence validating the consistency of the construct. Tourist Acceptance of Innovative Foods (TAIF) had the highest average values (m ≈ 5.4–5.7), which implies that the tourists in the sample are significantly willing to try, accept and recommend innovative dishes on the trip. Standard deviations were average (1.0–1.2), and the external loadings were given between 0.748 and 0.806, and this gave satisfactory convergent validity and internal consistency of this new construct. All in all, the findings validate that all indicators are valid measuring instruments of their latent constructs. All the external loads are more than the recommended 0.70 threshold, moderate and average values are theoretically meaningfully interpreted, and moderate standard deviations show the existence, but not extreme, expression of individual differences among tourists (Table 4).

The confirmatory factor analysis (CFA) outcomes obtained within the framework of the PLS-SEM revealed good values of reliability and validity of all constructs (Table 4). All of the constructs had Cronbach α values that were greater than the recommended value of 0.70, and this confirmed the internal consistency of the scales. Besides that, a measure of ρA (Rho_A) was also involved, which is a more conservative measure of reliability and more robust than α, and all values were obtained larger than the recommended number 0.70. The composite reliability (CR) was between 0.85 and 0.90, and the average variance extracted (AVE) was more than 0.50 in all constructs, which affirms convergence validity. A generation-by-generation comparison revealed some differences. The values of α, ρA, CR and AVE were a little bit more in the Baby Boomers group and can be attributed to the fact that the attitudes of older respondents towards food and gastronomy are more stable and consistent. In Generation Z, all thresholds were satisfied, but the values of AVE were in the lower limit, which indicates that this generation has more heterogeneity of attitudes and variation in behavioral patterns. The results show that the younger generation is more variable in terms of acceptance of innovative food and participating in gastronomy, and the answers of the older generation always are more consistent and stable (Table 5).

Although a preliminary exploratory factor analysis (EFA) confirmed the appropriateness of the data for factor extraction (KMO = 0.892; Bartlett’s test *p* < 0.001) and extracted one factor with an eigenvalue greater than 1 (1.433), that factor explained only 9.6% of the total variance. At first glance, this value may seem low, but additional confirmatory factor analysis (CFA) showed that the construct retains pronounced unidimensionality and internal consistency, as all factor weights exceeded 0.70, with CR = 0.88 and AVE = 0.54. It is important to emphasize that the construct TAIF (Tourist Acceptance of Innovative Food) is theoretically designed as a narrowly defined behavioral indicator that includes a specific aspect of tourists’ willingness to accept food innovations and not a broad domain of attitudes. In such cases, lower explained variance does not indicate a lack of reliability but rather conceptual precision and homogeneity of the construct. Similar findings have been confirmed in previous research in the field of consumer behavior and tourism, where constructs with a strong theoretical orientation and a smaller number of particles usually explain a smaller part of the variance but retain high factor weights and stable reliability indicators. Therefore, despite the more modest percentage of explained variance in EFA, the combination of CFA results, reliability indicators and theoretical justification confirms the validity and statistical stability of the TAIF construct within the SEM model. This interpretation is in line with modern methodological recommendations according to which theoretical coherence and convergent validity are more reliable indicators of construct validity than the explained variance itself.

### 4.2. Structural Equation Model Findings

Collinearity analysis of the internal coefficients revealed that none of the values of the coefficients of VIF exceeded the recommended value of 3.3, which means that there is no problem of multicollinearity in the model. This proved that the predictors play a role in the explanation of the dependent variable without regard to one another, and the estimated effects can be regarded as being steady and dependable [68]. The SRMR value of less than 0.08, which is below the recommended 0.08 value, was used to confirm that the global fit of the model was good. According to these parameters, it can be determined that the model is stable and statistically appropriate, which allows us to test hypotheses with structural relationships.

Table 6 results indicate that the addition of the FNS × FIS interaction has a slight yet significant increase in the explained variance of the dependent variable TAIF. The R^2^ of the base model was 0.546, and the extended model was 0.563, which is an extra ΔR^2^ = 0.017 (1.7% points of the explained variance). Even though the gain is not that high, it proves that the moderation effect does exist and helps to understand consumer behavior in a better way. Moreover, the Q^2^ value rose to 0.293 as compared to 0.278, which means that the extra model did not only explain the data but also was more predictively relevant than the base model.

The SEM analysis results prove that the negative and statistically significant impact of FNS on TAIF (β = –0.312, *p* < 0.001) proves that a stronger influence of neophobia on the willingness of tourists to accept innovative food takes place (H1). On the other hand, the significant and positive impact (β = 0.451, *p* < 0.001) is observed on FIS, which proves that increased food involvement leads to openness and innovative dishes should be proposed (H2). The moderating effect of FNS × FIS is also statistically significant (β = −0.087, *p* = 0.033), which supports hypothesis H3 and shows that high food involvement moderates the adverse effects of neophobia. Although the interaction effect (H3: FNS × FIS) is statistically significant (*p* = 0.033), the increase in explained variance (ΔR^2^ = 0.017) and the small effect (f^2^ = 0.021) indicate that the effect is of moderate intensity. This is theoretically consistent with consumer behavior research showing that psychological moderators such as food involvement rarely produce large statistical effects but play an important role in the fine regulation of behavior. Therefore, the significance of this interaction should not be seen through its magnitude but through its conceptual contribution, confirming that food involvement partially moderates the inhibitory effect of neophobia on tourists’ willingness to accept innovative food, in line with the PMT and TPB frameworks.

Effects of size (f^2^) indicate that the effect of FIS is medium (0.146), FNS us small to medium (0.082) and the interaction has a small but significant effect (0.021). The R^2^ (0.563) and Q^2^ (0.293) values also support the idea that the model is excellent in the explained variance and predictive relevance (Table 7).

**Figure 3:**
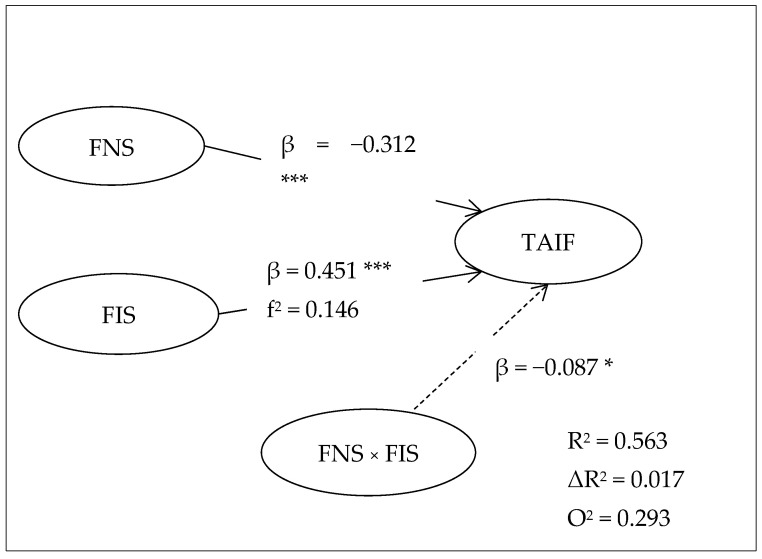
SEM results. Note: * *p* < 0.05; *** *p* < 0.001.

The results shown in Figure 3 clearly illustrate the relationships between the constructs and confirm the hypotheses about the influence of neophobia and food involvement on the acceptance of innovative gastronomic offers. Although the model shows satisfactory explained variance (R^2^) and predictive relevance (Q^2^), in order to further evaluate its out-of-sample predictive ability, a PLSpredict analysis was performed. This procedure provides a more robust evaluation of the model’s predictive power, comparing PLS predictions with a benchmark linear model (LM).

An additional check of the predictive power was carried out using the PLSpredict procedure in the program SmartPLS 4. The results indicate that the PLS-SEM model achieves superior out-of-sample predictions compared to the benchmark linear model (LM). For all constructs, Root Mean Squared Error (RMSE) and Mean Absolute Error (MAE) values were lower in the PLS-SEM model compared to LM, while all Q^2^_predict values were positive, which confirms the existence of predictive relevance out of the sample. This further confirmed that the model not only reliably explains the data from the sample but also has a stable predictive ability in a wider context (Table 8).

### 4.3. Measurement Invariance and Multi-Group Analysis Results

The results of the MICOM procedure confirmed that the model meets the conditions of measurement invariance in both generation groups. Configurational invariance was confirmed because the same model, the same indicators and the same assessment algorithm were used in both groups. The results of the permutation test for compositional invariance showed that all correlations between the original and permuted values of the constructs were close to 1 and statistically insignificant (*p* > 0.05), which confirmed that the constructs are formed in the same way in both groups. Finally, the test of equality of means and variances also showed no significant differences (*p* > 0.05), which means that there is complete measurement invariance. Based on these findings, it is justified to conduct a multigroup analysis (MGA) (Table 9).

The results of the Multi-Group Analysis indicate that there are significant generational differences in the factors shaping the acceptance of innovative food among tourists. The path FNS → TAIF (negative impact of food neophobia on acceptance) showed that the effect was statistically significantly stronger in Boomers (β = −0.402; *p* < 0.001) compared to Gen Z (β = −0.281; *p* < 0.001). This implies that older tourists show more pronounced reservations towards gastronomic novelties, which reduces their willingness to accept new tastes and concepts in hospitality. On the other hand, the path FIS → TAIF (positive impact of food involvement on acceptance) was significantly stronger in Gen Z (β = 0.496; *p* < 0.001) compared to Boomers (β = 0.379; *p* < 0.01). This result reflects the younger generations’ propensity for gastronomic innovation and willingness to experiment, which confirms their role as change agents in the digital and gastronomic transformation of the hospitality industry. These findings support the claim that the effect of neophobia on acceptance is more pronounced in groups with more traditional behavioral patterns (Boomers), while the role of food as a cultural and experiential resource is more strongly recognized in younger generations (Gen Z). Such differences have important implications for tourism and hospitality managers, as they point to the need for market segmentation and the design of gastronomic offerings in accordance with generational characteristics (Table 10).

## 5. Discussion

Even though all the hypotheses concerning the models have been confirmed, it is not possible to see these results as ones that are self-evident. The reliability of the results instead shows the theoretical strength of the suggested framework because both inhibitory (neophobia) and motivational (food involvement) processes are working in accordance with the assumptions of the Protection Motivation Theory (PMT) and the Theory of Planned Behavior (TPB). Nevertheless, the fact that the effects of innovation differ in strength across generations means that psychological responses to innovation cannot be universal. The findings clearly prove that the adoption of innovative food in tourism is determined by the dynamic relations between psychological objections and motivational factors. The adverse outcome of food neophobia was found to be consistent and statistically significant in both generational groups but stronger among representatives of the Baby Boomer generation. This observation is consistent with the fact that older consumers are characterized by more reservations toward innovation and an inclination to adhere to old and conventional eating habits, as has been shown in the works of Sui et al. [33] and Wiangkham et al. [34]. The current research builds upon these findings as it confirms that the effect of neophobia on the inhibitory dimension has a more significant impact in the setting related to innovative food, in which deep-rooted cultural eating behaviors complicate the process of adopting new eating habits in the gastronomic realm. This confirmed H1 but also showed that generational factors increase the intensity of this influence.

In spite of the fact that H1 is proven, demonstrating the existence of a negative and statistically significant impact of neophobia on the willingness of tourists to accept innovative food, it should also be mentioned that this correlation can be contextual. The strength of such an effect may be lower or even statistically non-significant in destinations where innovation is part of the culture or where there is greater reliance on institutions that manage food safety. In addition, neophobia can lose its inhibitory effect in those cases when innovations are marketed using forms that are familiar to locals (for example, fusion of traditional dishes). This means that the evidence for H1 is relative and is determined by cultural and economic background and experience, which leaves room for possible future research in other settings.

The generational gap that was revealed in the study shows that there is an in-depth socio-cultural aspect of food behavior. The greater negative influence of neophobia in Baby Boomers is indicative of a greater preference for stability and tradition, common to cohorts socialized in less diverse culinary conditions. Their hesitation toward innovation is in line with the research findings of Zhan et al. [9], who noted that perceived risk increases with age and cultural attachment. Generation Z, on the other hand, views food as an identity-centered experiential resource and not merely as a functional product. This aligns with the findings of Hossain et al. [8], who observed that younger consumers are more likely to incorporate innovation and cultural meaning in their tourism experiences. Thus, the gaps found between the two generations affirm that openness to innovation is not solely a personality aspect but also an outcome of socialization style, way of life, and exposure to global food trends.

Conversely, the results related to food involvement reveal a strong positive effect on the willingness to accept an innovative gastronomic offer, especially among Generation Z. This finding provides additional evidence in favor of H2 and builds on the research of Laureati et al. [17] and Samaddar and Mondal [20], who indicated that curiosity and balancing between the traditional and the innovative encourage sustainable tourist behavior. Our findings extend their argument by showing that the younger generation sees food not only as a food product but as a part of identity, social interactions, and experiential tourism, which was previously highlighted by Poyoi et al. [32]. This confirms that the motivational power of food is stronger among younger generations who are more open to experimentation and more ready to connect innovation with sustainability values. These results further confirm hypotheses H4 and H5, as they show that the generational framework shapes how both negative and positive dimensions of innovative food acceptance are manifested [28,31].

The special value of this research is that the integration of FNS and FIS in one model allows for a precise assessment of the balance between limiting and stimulating factors. Previous research has often focused on individual aspects, either on barriers related to neophobia [25,41] or on motivational dimensions related to the involvement and value of gastronomic experiences [40,43]. Our study shows that precisely their combination offers a deeper understanding of the acceptance of innovative food because tourists can be simultaneously curious and engaged but at the same time wary of the new and unknown. The results confirm that food involvement moderates the negative effect of neophobia on the acceptance of innovative foods. This finding indicates that tourists with high involvement, despite their expressed neophobia, remain more open to trying new gastronomic offers. In other words, motivation and engagement toward food can act as a protective factor that weakens the inhibitory influence of neophobia. Such results fit the works of Laureati et al. [17] and Samaddar and Mondal [20], who pointed out that curiosity and balancing between traditional and innovative encourage responsible consumer behavior. Our contribution lies in the empirical confirmation of this process in the tourism context, which additionally explains why certain groups of tourists are more ready to accept gastronomic innovations despite the existing barriers.

The uniqueness of the study is reflected in the fact that, for the first time, it combines food neophobia and food involvement as complementary psychological constructs in the analysis of the acceptance of innovative food, simultaneously considering generational differences and the perception of destination sustainability. While previous works analyzed innovations in the broader context of tourism, most often through technological services or cultural factors [14,15], this study offers a specific framework applied to gastronomy as an integral part of the tourist experience. This opens up space for a new understanding of how innovative food can contribute to the sustainability and competitiveness of destinations but also for the development of managerial strategies that differentiate the offer in accordance with the psychological profiles of different generations.

The established generational contrast can be seen as a major theoretical contribution to the process of defining tourist behavior as the joint expression of motivational and barrier dimensions. It has been confirmed empirically that psychological constructs like neophobia toward food and participation in food are not universal but are situational, based on larger cultural and generational contexts. The given knowledge broadens the current theories of behavior by demonstrating that the inclination to accept innovations in the gastronomic area is predetermined by the value orientations and the patterns of socialization peculiar to a particular generation.

## 6. Conclusions

This study showed that the acceptance of innovative food in tourism is shaped through complex dynamics between psychological barriers and motivational drivers, but also through destination-specific contextual conditions. In Serbia, where the tourism sector still faces the consequences of limited international recognition, a weaker tradition in shaping the gastronomic offer, and reduced travel mobility due to economic and geopolitical circumstances, innovative food appears as a special tool for differentiation and shaping authentic experiences. Our results show that, although resistance to novelties remains pronounced among older tourists, the motivation and openness of younger generations open the possibility for gastronomic innovations to become a key part of the tourism development strategy. This confirms that food is not only a segment of the offer but also a strong cultural and symbolic resource of the destination.

### 6.1. Theoretical Implications

The original contribution of this work is reflected in the fact that the integration of the constructs of food neophobia and food involvement in one model not only opens a new perspective for understanding the acceptance of innovations in tourism but also theoretically fits into the specific context of countries in transition. In Serbia, where tourism continues to balance between the inherited patterns of the post-socialist society and the contemporary trends of the global industry, this integration shows how psychological factors shape the process of accepting innovations within a culturally and economically dynamic framework [72]. In this way, the study contributes to the theory of innovations in tourism by demonstrating that innovative food in transition economies functions not only as a means of differentiation but also as a testing ground for re-examining the relationship between tradition and modernity. This opens up the possibility of developing a theoretical framework of “gastronomic transition” that connects psychological, cultural, and developmental dimensions and can be applied to other post-transition destinations.

### 6.2. Practical Implications

In the context of Serbia as a country in transition, the practical implications of this research go beyond the standard recommendations on market segmentation and adjustment of the offer to generations [73]. A destination that continues to face a weak international image, dependence on political and economic fluctuations, and insufficiently developed branding can find a stable differentiator based on internal resources and creative capital precisely through innovative food. Such a strategy allows gastronomy to be transformed from a traditional symbol into a dynamic instrument that simultaneously communicates authenticity and openness to the new [74]. For younger tourists, innovative food can shape the image of Serbia as a modern and sustainable destination that follows global trends, while for older visitors, the reinterpretation of familiar dishes in an innovative key reduces resistance to change and encourages loyalty to the destination [72]. In this way, gastronomic innovations are not only an attraction for visitors but also a strategic tool for redefining Serbia’s tourist identity, strengthening competitiveness in the international market, and creating self-sustainable development independent of external political and economic circumstances. It is necessary to add that the sustainability attitudes were not a direct part of the PLS-SEM model. As such, the analysis of the results with respect to sustainable gastronomic tourism should be viewed in terms of interpretive and theoretical significance and not as one that is empirically tested. These findings, however, indicate that the receptiveness of the younger generations to innovation can become a behavioral basis of sustainability-focused tourism practices, which needs to be further examined by directly incorporating sustainability constructs in subsequent models.

### 6.3. Limitations and Future Research

Despite the fact that the results contribute to the critical knowledge in the context of the adoption of innovative food, there are some limitations of the research that should be taken into account. Only domestic tourists in Serbia were used as a sample. Whereas it offers an insightful idea about the local trends in the behavior and habits of a nation with a poorly established culture of gastronomic tourism and low mobility of people via road, the results cannot be fully extrapolated to foreign tourists, for whom innovative food can be a totally different value and cultural aspect. During the research period, from December 2024 to June 2025, a number of political and economic challenges were recorded in the country, which led to a significant decrease in the number of foreign tourists, especially in larger urban and tourist centers. In such circumstances, the inclusion of a limited number of foreign visitors in the sample would not be methodologically justified, as it would create an imbalance between the populations and reduce the validity of the findings. Therefore, the focus on domestic tourists enabled a more reliable and representative assessment of attitudes and behavior in the national context. Additionally, the representativeness might have been affected by the proper sampling technique, and the sample composition (e.g., uneven generation ratio or other prevalence of some of the sociodemographic groups) might have predetermined the results in a certain direction.

Moreover, the study was based on self-reports gathered via a survey, and it opens the prospects of respondent bias and the influence of socially desirable answers. Though the scales (FNS and FIS) and a standardized seven-point Likert format were validated, such a methodology measures the perceptions and intentions, not the actual behavior. The cross-sectional design also restricts the possibility to establish cause-and-effect relationships and trace the evolution of attitudes with time.

Despite the fact that the article Food Neophobia and Food Involvement has offered a solid model on the psychological processes, studies have concentrated on two dimensions. The other pertinent features that may have contributed to the complexity of gastronomic innovations adoption—cultural curiosity, emotional intelligence, risk perception, or sustainability attitudes—were not included, yet these may also be relevant. In addition, the generational comparison was narrowed down to the members of Generation Z and Baby Boomers, whereas other groups of people (Millennials or Gen X) were excluded, and this might make a more comprehensive picture in the future. One of the main limitations of this research is related to generational coverage. The research was focused exclusively on Generation Z and Baby Boomers, in order to highlight the contrast between the youngest and oldest consumer groups. Although such an approach allowed deeper insights, the exclusion of Generations X and Y limits the possibility of generalizing the findings to the entire adult population. Future research should include Millennials and Generation X to capture intermediate behavioral patterns and provide a more comprehensive understanding of innovation acceptance among different age groups.

Furthermore, the question of the unfinished responses in the questionnaires is also one of the limitations. The total sample size consisted of 985 respondents, but only complete questionnaires (listwise deletion) were factored and structurally analyzed (EFA, CFA, SEM, MGA), which contributed to a slight disparity in the number of respondents per analysis. Nevertheless, any successful subsamples were still significantly above the lowest methodological standards and, therefore, did not impact the stability of the results and their validity. The other weakness associated with this study is related to the lack of sustainability attitudes as a construct in the PLS-SEM model. This has been performed so as to maintain the conceptual simplicity and ease of the model, though it restricts the potential of empirically testing the relationship between innovation and sustainable tourist behavior. Sustainability variables should also be incorporated in future studies to enhance the knowledge of psychological and motivational factors that can be used to achieve sustainable gastronomic practices.

In this study, the principle of model parsimony was applied, given that two fundamental psychological determinants, food neophobia and food involvement, were considered, and other possible determinants, including cultural curiosity, emotional intelligence, risk perception, and sustainability attitudes, were deliberately left out. This was performed to give conceptual clarity and statistical stability to the model at an earlier point in testing. However, future studies can broaden this framework and incorporate the mentioned constructs, particularly attitude towards sustainability, which has already been addressed in the theoretical segment of the paper and which may help to make the theoretical aspect of the model more profound and explanatory. Even though interviewer instructions were standardized and pilot-tested, the fact of slight differences in the understanding of the concept of innovative food by respondents cannot be altogether excluded. This should be remedied in future studies through giving visual illustrations or experimental stimuli under control to aid in efforts of improving the precision of measurements.

In addition, the short, one-dimensional form of the Food Involvement Scale (FIS) might not have captured refined details of consumer involvement in regard to particular phases of food behavior (purchase, preparation, and consumption). It is recommended that future research undertakings be performed to test the entire multidimensional version of the FIS in order to be able to capture these less obvious forms of involvement.

It is suggested that this could be extended to international tourists in the future to observe the variation in the perception of innovative food among tourists who are already familiar with the domestic cuisine and those who are entirely new to the same in the future. Future research should expand the scope of the model by including international tourists, as well as testing reactions to completely unfamiliar, exotic, or foreign foods. In this way, it would be possible to examine how cultural distance, sensory unknowns and different levels of gastronomic curiosity affect the intensity of neophobia and the willingness to accept innovative food. This approach would enable a deeper understanding of the cultural dimension of gastronomic innovations and contribute to the formation of comparable findings among different markets and destinations. Longitudinal design might help illuminate the shifts in attitude and behavior throughout the years, particularly when issues of global concern like crises, migration, or lifestyle changes are involved. Also, it would be possible to add qualitative techniques (e.g., in-depth interviews or focus groups) to gain a better insight into cultural and emotional influences on perceptions of food innovations. Lastly, the comparisons between various destinations and industries would make it possible to create a more comprehensive understanding of gastronomic innovation that would bring benefits to theory as well as contribute to the formulation of practice in tourism on the global scale.

## Figures and Tables

**Table 1:** Generational structure of the sample.

Generation	Years of Birth	Label in Analysis	Key Characteristics
Baby Boomers	1946–1964	Older cohort	Tradition-oriented, cautious toward innovation
Generation Z	1995–2010	Younger cohort	Tech-savvy, experimental, innovation-oriented

**Table 2:** Sociodemographic characteristics of tourists.

Variable	Category	Gen Z (*n* = 482)	Boomers (*n* = 503)	Total
Gender	Male	224	262	486
	Female	258	241	499
Age	1995–2010	482	–	482
	1946–1964	–	503	503
Education	High school	156	198	354
	College/Higher	305	271	576
	PhD	21	34	55
Income (€)	<500	188	241	429
	500–1000	224	201	425
	1000+	70	61	131
Employment	Student	162	0	162
	Employed	258	87	345
	Unemployed	34	29	63
	Retired	28	387	415
Type of travel	City tourism	248	187	435
	Spas and wellness	96	164	260
	Mountain resorts	138	152	290

Source: Author’s research.

**Table 3:** Measurement constructs, sources and items.

Constructs	Source	Statements (Likert 1–7)
Food Neophobia Scale (FNS)	Pliner & Hobden [20]	FNS1: I don’t like to try food that I have never eaten before. FNS2: I avoid dishes that seem too unfamiliar to me. FNS3: I am wary of unusual foods. FNS4: I prefer to choose foods that I am already familiar with. FNS5: New foods make me distrustful.
Food Involvement Scale (FIS)	Bell & Marshall [23]	FIS1: Food occupies an important place in my life. FIS2: I often think about food during the day. FIS3: I like to prepare and experiment with food. FIS4: When I travel, I always want to try local dishes. FIS5: I enjoy discovering new restaurants and tastes.
Tourist Acceptance of Innovative Foods (TAIF)	Ajzen [57], Kim et al. [58]	TAIF1: I am willing to try innovative dishes while traveling. TAIF2: If I am offered a new dish, I will try it without hesitation. TAIF3: I would accept plant-based or fusion dishes in a hotel/restaurant. TAIF4: I would recommend innovative dishes to other tourists. TAIF5: I accept innovative food as part of the tourist experience.

Source: Author’s research.

**Table 4:** Descriptive metrics and outer loadings.

Construct	Item	m	sd	λ
FNS	FNS1	3.842	1.104	0.734
	FNS2	3.765	1.089	0.768
	FNS3	3.921	1.147	0.751
	FNS4	4.012	1.215	0.783
	FNS5	3.876	1.093	0.759
FIS	FIS1	5.214	1.042	0.721
	FIS2	5.031	1.087	0.706
	FIS3	4.983	1.192	0.744
	FIS4	5.341	1.153	0.762
	FIS5	5.148	1.083	0.737
TAIF	TAIF1	5.672	1.062	0.781
	TAIF2	5.489	1.108	0.764
	TAIF3	5.415	1.172	0.748
	TAIF4	5.608	1.125	0.792
	TAIF5	5.534	1.137	0.806

Source: Author’s research.

**Table 5:** Reliability and convergent validity of constructs.

Construct	α	ρA	CR	AVE
Total (*N* = 985)				
FNS	0.841	0.86	0.879	0.592
FIS	0.827	0.85	0.866	0.568
TAIF	0.860	0.87	0.890	0.621
Gen Z (*N* = 563)				
FNS	0.819	0.84	0.870	0.575
FIS	0.805	0.83	0.857	0.552
TAIF	0.851	0.87	0.885	0.613
Boomers (*N* = 422)				
FNS	0.848	0.87	0.886	0.609
FIS	0.835	0.86	0.874	0.583
TAIF	0.870	0.89	0.903	0.645

Note: α—Cronbach’s Alpha, ρA—Rho_A conservative estimate of reliability, CR—Composite reliability, AVE—Average variance extracted: (α, ρA, CR ≥ 0.70; AVE ≥ 0.50). Source: Author’s research.

**Table 6:** Comparison of predictive power (ΔR^2^ and Q^2^).

Model	R^2^ (TAIF)	ΔR^2^	Q^2^ (TAIF)
Basic (no interaction)	0.546	–	0.278
+ Interaction FNS × FIS	**0.563**	**0.017**	**0.293**

Source: Author’s research. Note: Bold values indicate an improvement in predictive power

**Table 7:** Hypothesis test outcomes.

Hypothesis	Path	β	SE	t	*p*	f^2^	R^2^	Q^2^	Confirmation
H1	FNS → TAIF	–0.312	0.058	5.379	<0.001	0.082			confirmed
H2	FIS → TAIF	0.451	0.062	7.274	<0.001	0.146			confirmed
H3	FNS × FIS → TAIF	–0.087	0.041	2.134	0.033	0.021	0.563	0.293	confirmed

Note: f^2^—effect size (≈ 0.02 (small), 0.15 (medium), 0.35 (strong)); β—beta coefficient, SE—standard error, *t*-test statistic for hypothesis testing (t ≠ 0), *p*—probability value (*p* < 0.05). Source: Author’s research.

**Table 8:** PLS predict results (out-of-sample predictive power).

Construct	RMSE (PLS)	RMSE (LM)	MAE (PLS)	MAE (LM)	Q^2^_Predict
FNS	0.912	0.954	0.721	0.748	0.214
FIS	0.875	0.902	0.695	0.713	0.239
TAIF	0.843	0.881	0.668	0.689	0.267

Note: RMSE—Root Mean Squared Error; MAE—Mean Absolute Error; Q^2^_predict > 0 indicates predictive relevance beyond the sample. Source: Author’s research.

**Table 9:** Measurement invariance of composite models.

Step	Test	Result	Permutation *p*-Value	Conclusions
1	Configural invariance	Satisfied	–	Identical setup confirmed
2	Compositional invariance	Correlation = 0.996	0.432	Invariance established (*p* > 0.05)
3a	Equality of means (FNS)	ΔMean = 0.021	0.287	Equal across groups
3b	Equality of variances (FNS)	ΔVar = 0.018	0.351	Equal across groups
3a	Equality of means (FIS)	ΔMean = 0.034	0.298	Equal across groups
3b	Equality of variances (FIS)	ΔVar = 0.027	0.410	Equal across groups
3a	Equality of means (TAIF)	ΔMean = 0.016	0.365	Equal across groups
3b	Equality of variances (TAIF)	ΔVar = 0.022	0.389	Equal across groups

Note: All *p*-values > 0.05, confirming full measurement invariance across Gen Z and Boomer groups. Source: Author’s research.

**Table 10:** MGA results.

Path	Gen Z (β)	Boomers (β)	Δβ	*p*-Value	Conclusions
FNS → TAIF	−0.281	−0.402	0.121	0.018	stronger negative effect in Boomers
FIS → TAIF	0.496	0.379	0.117	0.031	stronger positive effect in Gen Z

Source: Author’s research.

## Data Availability

The original contributions presented in the study are included in the article, further inquiries can be directed to the corresponding author.
